# Giant cell arteritis without cranial manifestations caused mesenteric involvement: a case report

**DOI:** 10.1186/s40792-019-0678-6

**Published:** 2019-07-24

**Authors:** Yasuhiro Miyake, Yoshikazu Morimoto, Masaaki Taniguchi, Chihiro Yamanaka, Goro Ueno, Sakae Ejima, Chikao Yutani

**Affiliations:** 1 Department of Surgery, Japan Community Healthcare Organization (JCHO) Osaka Bay Central Hospital, 1-8-30 Chikko, Minato-ku, Osaka, 552-0021 Japan; 2 Department of Pathology, Japan Community Healthcare Organization (JCHO) Osaka Bay Central Hospital, 1-8-30 Chikko, Minato-ku, Osaka, 552-0021 Japan

**Keywords:** Giant cell arteritis, Mesenteric involvement, Extracranial manifestations

## Abstract

**Background:**

Giant cell arteritis (GCA) is a granulomatous vasculitis and targets large vessels with predominance for the aortic arch and the cranial branches. GCA with cranial symptoms shows headache, jaw claudication, and ophthalmologic symptoms and thus was previously called temporal arteritis. Recently, cases of GCA without cranial manifestations and extracranial GCA have been reported.

**Case presentation:**

A 76-year-old woman was referred to our hospital complaining of sudden abdominal pain and high fever. Her present history of illness did not show any cranial symptoms such as headache, visual disturbance, or stroke. CT images showed severe thickening of the small intestinal mesentery and massive ascites. She was diagnosed to have acute abdomen probably with gastrointestinal perforation and underwent the emergent laparotomy. Excisions of a 60-cm length of the jejunum including the thickening mesenteric lesion were carried out. Marked hypertrophy of the vascular intima and mild stenosis of the arterial lumen were displayed with infiltration of lymphocytes, neutrophils, and eosinophils. Scattered multinucleated giant cells on the endothelium, in the intima, media, and adventitia were demonstrated. Elastica van Gieson stain showed focal loss and fragmentation of the internal elastic lamina. Histopathological examinations showed typical GCA. Her postoperative process was uneventful without any symptoms, and she was followed as an out-patient prescribed with daily doses of 40 mg of prednisolone.

**Conclusions:**

We hereby report a rare case of mesenteric involvement in GCA without cranial manifestations and elucidate the histopathological features of extracranial GCA in arteries as well as veins and jejunum.

## Introduction

Giant cell arteritis (GCA) is a well-known form of vasculitis, affecting large vessels including aortic arch and cranial branches. GCA with cranial symptoms (i.e., headache, jaw claudication, ophthalmologic symptoms, and clinical temporal artery anomaly) is frequently reported and was previously called temporal arteritis [1–5]. However, mesenteric involvement in GCA without cranial presentation is rare; thus, further study of this clinical entity should be required. We report a case of GCA without cranial manifestation in a patient who presented an acute abdomen due to mesenteric involvement.

## Case presentation

A 76-year-old woman who presented with sudden abdominal pain and high fever was referred to our hospital. On physical examination, her abdomen was distended with rebound tenderness and no bowel sounds were audible. Body temperature was 38.0°, pulse rate 100/min, right arm blood pressure 93/57 mmHg, and left arm blood pressure 89/65 mmHg. In her present history of illness, she denied cranial symptoms such as headache, visual disturbance, or stroke. She had a history of renal stones and had undergone abdominal contrast-enhanced computed tomography (CT) scan 5 years before. The CT images showed no remarkable findings of abdominal large vessels.

**Fig. 1:**
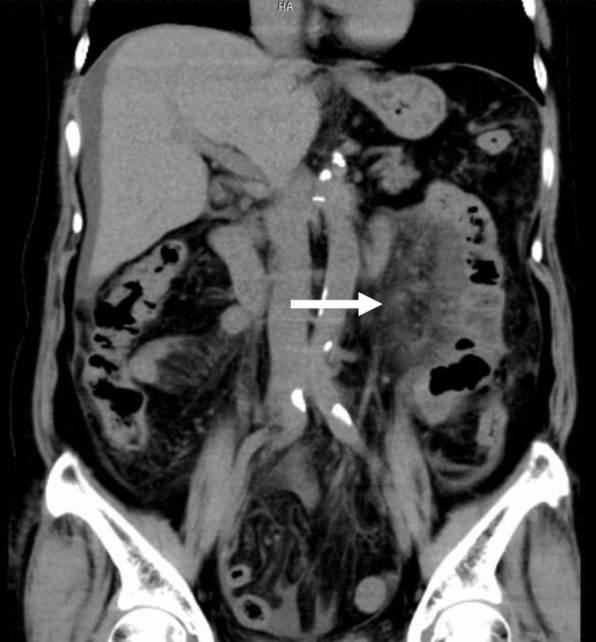
CT image on admission. The thickening mesentery adjacent to the jejunum is showed (arrow). A periarterial halo or stenosis in the superior mesenteric artery is not remarkably visualized

Laboratory data on admission showed a high level of C-reactive protein (CRP) and white blood cell counts (WBC), 17.75 mg/dl and 11.4 × 10^3^/mm^3^, respectively. Liver and renal functions were normal. CT images showed severe thickening of the small intestinal mesentery and massive ascites. However, a periarterial halo around the superior mesenteric artery was not revealed (Fig. 1). She was diagnosed to have acute abdomen probably with gastrointestinal perforation and underwent emergent laparotomy.

Laparotomy revealed no gastrointestinal perforation. Massive ascites was determined but was not dirty. The small bowel at 50 cm from the Treitz ligament was narrow and reddish with a length of 10 cm; the intestinal mesentery restricted to this area was severely thickening, and a hard mass of 4 cm in size was determined. Excisions of a 60-cm length of the jejunum including the thickening mesenteric lesion were carried out and reconstruction was done using functional end-to-end anastomosis.

### Histopathological examination

**Fig. 2:**
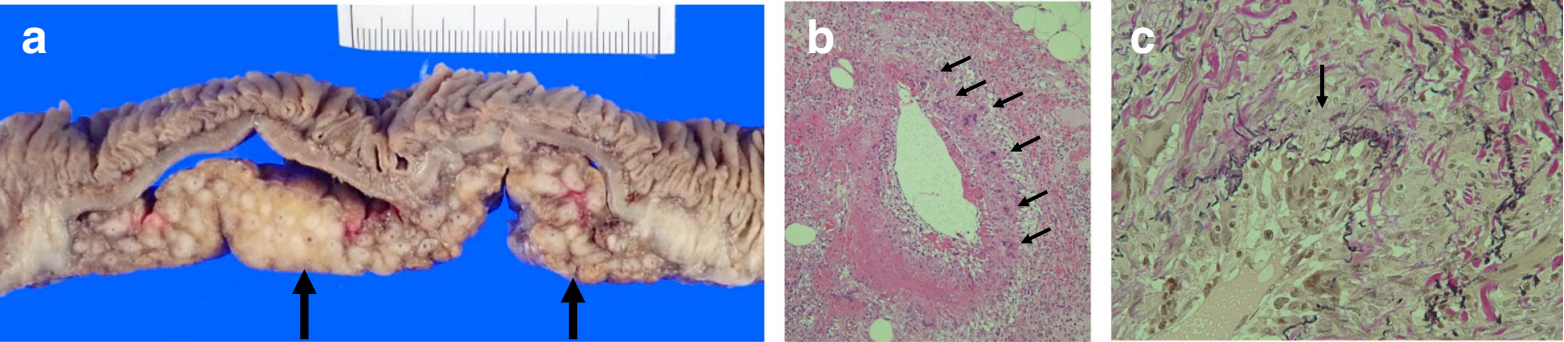
Histopathological examinations. **a** Specimen of the resected jejunum and mesentery: The mesentery includes significant nodular thickening (arrow). Each nodule has central canals composed of the vessel. **b** The arterial wall structure of GCA was broken, and all layers were not clear to distinguish. Hypertrophy of vascular intima is remarkable. Stenosis of the arterial lumen is mildly displayed. Multinucleated giant cells (arrow) are scattered on the endothelium, in the intima, media, and adventitia. Lymphocyte, neutrophils, and eosinophils are also infiltrated. **c** Elastica van Gieson stain highlights the internal elastic lamina, which demonstrates loss and fragmentations. Phagocytosis of elastic fibers is remarkably showed (arrow)

No perforation was found in the resected jejunum. The mesentery adjacent to the excised jejunum showed a significant nodular thickening with central canals which were composed of blood vessels (Fig. 2a). Marked hypertrophy of the vascular intima and mild stenosis of the arterial lumen were displayed with infiltration of lymphocytes, neutrophils, and eosinophils. Scattered multinucleated giant cells on the endothelium, in the intima, media, and adventitia were demonstrated (Fig. 2b). A special elastin stain showed focal loss and fragmentation of the internal elastic lamina. Phagocytosis of elastic fibers was remarkably showed (Fig. 2c). Immunohistochemical stains of CD68 on giant cells and CD8 on lymphocytes were positive (figures were not showed).

The postoperative process was uneventful besides paralytic ileus. She was discharged after 30 postoperative days and was followed as an out-patient prescribed with daily doses of 40 mg of prednisolone. She had neither abdominal symptom nor cranial manifestations and displayed lower levels of inflammatory laboratory parameters in CRP and WBC, except for a high level of erythrocyte sedimentation rate at 45 mm/h.

## Discussion

GCA is a granulomatous vasculitis of unknown origin and targets large vessels with predominance for the aortic arch and the cranial branches [1, 2, 6]. Most patients in GCA present cranial manifestations described above; thus, GCA was previously called temporal arteritis. GCA usually occurs in elderly women over the age of 50 in western countries, while the incidence of GCA in Asian is relatively rare compared to that of Takayasu’s arteritis [2].

Some authors have recently reported a few subgroups of GCAs [3, 7–10]. Hayreh et al. investigated 85 GCA patients, of which occult GCA, defined as ocular involvement without any systemic symptoms, occupied 21% [7]. De Boysson et al. presented 143 GCA patients, and GCA without cranial manifestations occupied 22% [3]. According to studies in GCA without cranial symptoms, the characteristics were described as follows: lower inflammatory laboratory parameters, more frequent large arterial involvement, and less disease relapse [3, 7, 8]. Therefore, GCA should be increasingly recognized as a systemic vascular disease even if patients have no typical cranial manifestations in GCA.

Recent studies have suggested that GCA quite often manifests exclusively in large arteries, i.e., the aorta and proximal branches, with specific symptoms frequently not present. This has been referred to as “extracranial” GCA [5, 10]. Extracranial manifestations due to GCA are not commonly reported, and only 9% of patients with GCA exhibit involvement of extracranial sites such as the thorax, abdomen, and pelvis [4]. The diagnosis of extracranial GCA can be elusive because of the frequent paucity of symptoms [11, 12].

Mesenteric ischemia in GCA, which is one of the extracranial GCA entities, was first described by Hamrin et al. in 1965 [13], and since then, only 30 patients have been reported in English and French literatures [1, 11, 14–16]. Scola et al. reviewed 12 cases of mesenteric involvement in GCA and showed that survival was observed in only 50% [16]. The authors suggest that mesenteric vasculitis, only rarely described in GCA, represents serious complication resulting in small bowel infarction [14–16]. Grayson et al. analyzed massive cohort studies and showed that the frequency of arteriographic lesions of the mesenteric artery in GCA was 18% [17] and thus reports of mesenteric involvement in GCA may increase in future.

We reviewed our patient’s clinical course. She denied cranial symptoms such as temporal scalp pain, visual disturbance, masseter pain, or stroke. Extremity claudication was not determined. We examined additional studies containing laboratory tests and positron emission tomography (PET) imaging. Complements C3 and C4, hepatitis B and C serologies, antinuclear antibodies, and antineutrophil cytoplasmic antibodies were at normal range. PET imaging studies did not show cranial GCA nor extracranial GCA, namely no remarkable GCA findings of the aorta and other main branches were determined. According to our patient’s past medical history, she had already undergone abdominal contrast-enhanced CT 5 years prior to this clinical episode, at 71. The CT images did not show any typical GCA characteristics at the mesenteric arteries; thus, the patient’s clinical entity of mesenteric involvement in GCA might have gradually occurred during these 5 years in her 70s. Although neither perforation nor small bowel infarction was found in our case, and she could have suffered life-threatening critical episode prior to the surgical intervention.

**Fig. 3:**
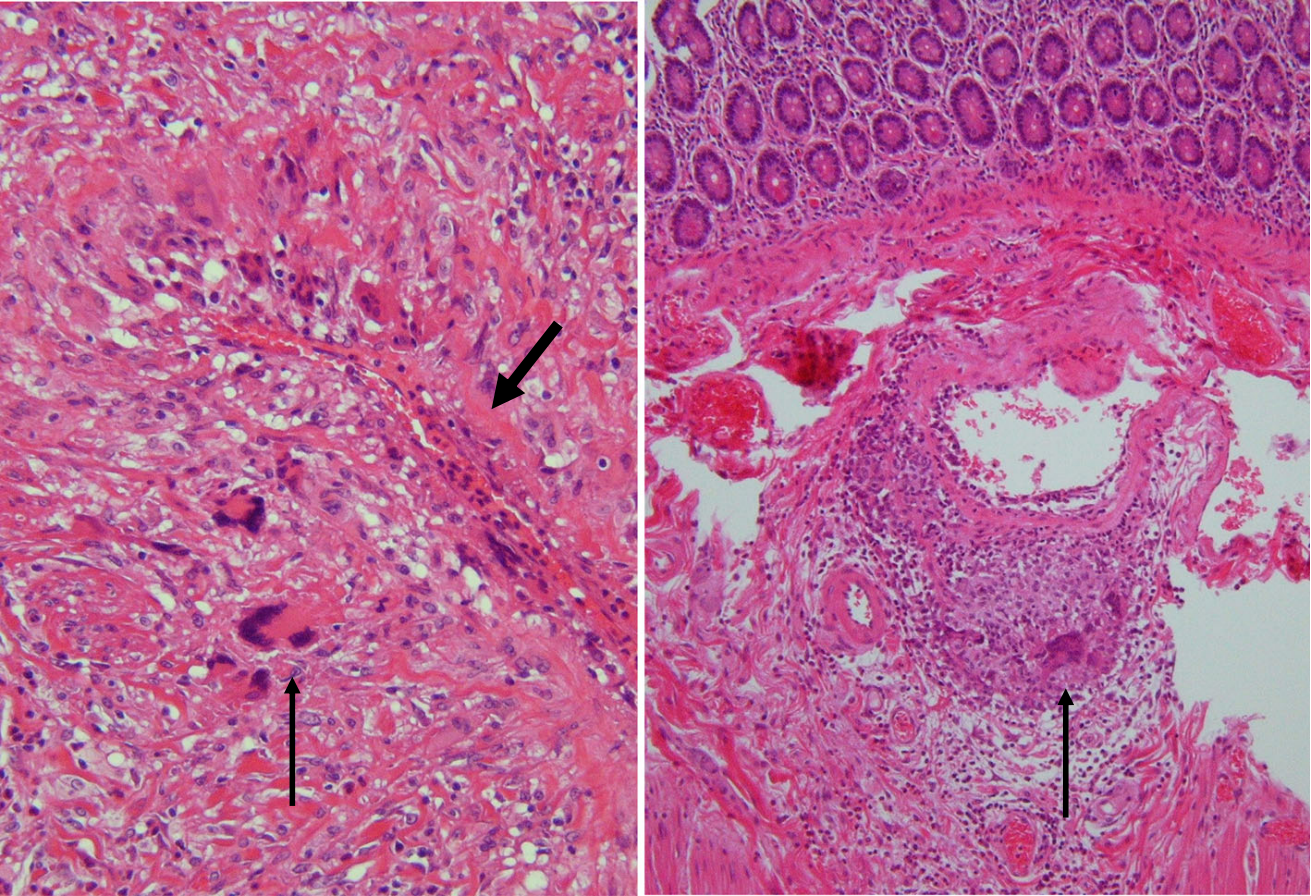
Histological examination in the area of the vein (left) and small intestine (right). The thickening wall of the vein is remarkable (thick arrow). Granulomatous infiltration of lymphocyte, neutrophils, and eosinophils as well as multinucleated giant cells (thin arrow) is viewed in both fields

Furthermore, we retrospectively studied our patient in histopathological finding. She presented an acute abdomen in extracranial GCA without any typical cranial symptoms. The thickening small bowel mesentery included a large mass, which was composed of small massive nodules and showed typical mesenteric vasculitis in GCA. The mesenteric tumor formation caused by GCA may have also induced massive ascites. Histological features also displayed intimal thickening and granulomatous inflammation, including giant cells, lymphocytes, and fragmented elastic lamina. In our case, these typical characteristics of GCA were determined in both the vein and the intestinal wall (Fig. 3). In histopathological, an excised specimen should be necessary to examine not only arteries but also veins and other organs for the patients with extracranial GCA.

## Conclusions

In conclusion, it should be considered that mesenteric involvement in GCA can occur without any typical cranial manifestations and might result in devastating clinical outcome.

## Data Availability

The datasets supporting the conclusions of this article are included within the article.

## Abbreviations

CRP: C-reactive protein
CT: Computed tomography
GCA: Giant cell arteritis
PET: Positron emission tomography
WBC: White blood cell counts
